# Rapid weight loss with dietary salt restriction in hospitalized patients with chronic kidney disease

**DOI:** 10.1038/s41598-019-45341-6

**Published:** 2019-06-19

**Authors:** Yu Mihara, Hiroshi Kado, Isao Yokota, Yayoi Shiotsu, Kazuhiro Sonomura, Tetsuro Kusaba, Tsuguru Hatta, Satoaki Matoba, Keiichi Tamagaki

**Affiliations:** 1 Department of Nephrology, Graduate School of Medical Science, Kyoto Prefectural University of Medicine, Kyoto, Japan; 2 Department of Nephrology, Omihachiman Community Medical Center, Shiga, Japan; 3 Department of Biostatistics, Graduate School of Medicine, Hokkaido University, Hokkaido, Japan; 4 Department of Cardiovascular Medicine, Graduate School of Medical Science, Kyoto Prefectural University of Medicine, Kyoto, Japan

**Keywords:** Oedema, Acid, base, fluid, electrolyte disorders, Chronic kidney disease, Outcomes research

## Abstract

Dietary salt restriction is essential for managing fluid retention in patients with chronic kidney disease (CKD). In this retrospective cohort study, we investigated weight loss from the perspective of fluid status in CKD patients during a 7-day hospitalization period while consuming a low-salt diet (5 g/day). Among 311 patients, the median weight loss (interquartile range, maximum) was 0.7 (0.0–1.4, 4.7) kg on Day 4 and 1.0 (0.3–1.7, 5.9) kg on Day 7. Patients were classified into quartiles based on pre-hospital urinary salt excretion (quartile [Q] 1, 1.2–5.7; Q2, 5.8–8.4; Q3, 8.5–11.3; Q4, 11.4–29.2 g/day). Weight loss was significantly greater in Q3 and Q4 than in Q1. The body mass index (BMI) and urinary salt excretion in the first 24 hours after admission were independently associated with rapid weight loss on Day 4 by multivariate logistic regression analysis. In conclusion, CKD patients with a high salt intake or high BMI exhibit rapid weight loss within a few days of consuming a low-salt diet. Dietary salt restriction is effective for reducing proteinuria in these patients, but long-term observation is needed to confirm the sustained effects.

## Introduction

Chronic kidney disease (CKD) can cause fluid retention, mainly due to reduced glomerular filtration of sodium and activation of the renin-angiotensin system. Many patients with stage 3–5 CKD present fluid retention, which may lead to a higher cardiovascular risk via several pathways^1–4^. First, fluid retention is related to hypertension in CKD patients and salt sensitivity of blood pressure increases with decreasing kidney function^5^. Hypertension associated with fluid retention in CKD patients often requires multiple medications^6^. Second, fluid retention causes endothelial oxidative stress and release of inflammatory mediators, which have been demonstrated to increase the cardiovascular risk^7^. In order to control fluid retention, salt restriction is essential. Therefore, the Japanese Society of Nephrology recommends restricting the salt intake to <6 g/day for CKD patients^8^, whereas the Kidney Diseases Improving Global Outcomes (KDIGO) guidelines recommend <5 g/day^9^.

In clinical settings, CKD patients with apparent fluid retention frequently experience rapid weight loss within a few days of consuming a low-salt diet during hospitalization. This decrease in body weight has been considered to occur due to reduced fluid retention. In the 1950’s, Strauss *et al*. reported that when salt intake was abruptly changed in healthy subjects, it took 3–4 days until a new steady state in which renal sodium excretion matched intake was achieved, and thus a negative salt balance contributed to weight loss^10–12^. Some clinical studies have reported weight loss within several weeks after dietary salt restriction^13–16^, whereas others did not observe significant weight loss with a longer observational period^17,18^. However, limited information is currently available on weight loss immediately after dietary salt restriction in CKD patients.

Therefore, we investigated the short-term effects of dietary salt restriction on weight loss from the perspective of fluid status in CKD patients. We also performed a multivariate logistic regression analysis to identify the factors related to rapid weight loss in response to dietary salt restriction.

## Results

### Participant characteristics

During the study period, 461 patients were assessed for eligibility. Of these, we excluded 115 patients who were missing data for body weight during hospitalization, 30 patients missing data for 24-hour urinary salt excretion, and 5 patients missing data for 24-hour proteinuria before or during hospitalization. As a result, 311 patients were included in the study. The clinical characteristics of study participants are listed in Table 1. The median age was 69 years, 74.3% were men, 47.9% had diabetes, and the median estimated glomerular filtration rate (eGFR) was 31.0 ml/min per 1.73 m^2^. The median pre-hospital 24-hour proteinuria was 0.50 g/day.

**Table 1:** Clinical characteristics of study participants. Data are shown as numbers (percentage) for categorical variables and the median (interquartile range) for continuous variables. Abbreviations: eGFR, estimated glomerular filtration rate.

Characteristics	Total (n = 311)
Age, years	69 (62–76)
Male, %	231 (74.3)
Diabetes, %	149 (47.9)
Diuretic use, %	106 (34.1)
Body mass index, kg/m^2^	23.9 (21.1–27.0)
24-hour systolic blood pressure, mmHg	125 (115–135)
24-hour diastolic blood pressure, mmHg	75 (69–81)
Serum albumin, g/dl	3.9 (3.6–4.2)
Serum creatinine, mg/dl	1.64 (1.23–2.50)
eGFR, ml/min per 1.73 m^2^	31.0 (19.1–44.5)
Hemoglobin, g/dl	11.9 (10.5–13.4)
Proteinuria, g/day	0.50 (0.11–1.81)

### Weight loss after admission

**Figure 1:**
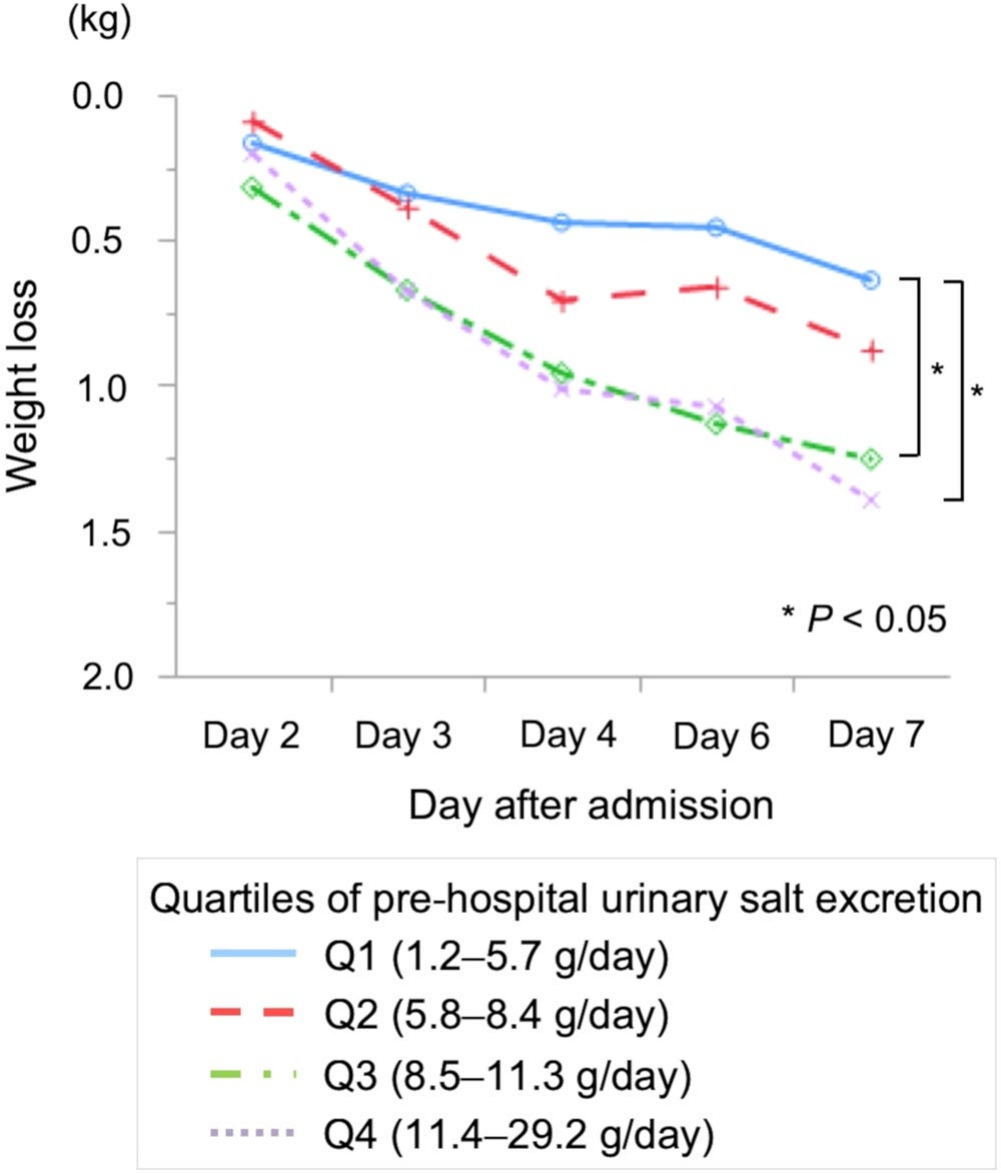
Weight loss after admission by quartiles of pre-hospital urinary salt excretion. Weight loss was significantly greater in the high urinary salt groups (Q3 and Q4) than in Q1 (*P* = 0.012 and 0.015, respectively). Abbreviations: Q, quartile.

Patients were classified into quartiles based on pre-hospital urinary salt excretion (quartile [Q] 1, 1.2–5.7; Q2, 5.8–8.4; Q3, 8.5–11.3; and Q4, 11.4–29.2 g/day). Weight loss after admission was significantly greater in the high urinary salt excretion groups (Q3 and Q4) than in Q1 by one-way analysis of variance (ANOVA) with post-hoc Tukey’s honestly significant difference (HSD) tests (*P* = 0.012 and 0.015 respectively; Fig. 1). The weight loss on Day 4 and Day 7 by quartiles of pre-hospital urinary salt excretion is shown in Table 2. Among all subjects, the median weight loss (interquartile range, maximum) was 0.7 (0.0–1.4, 4.7) kg on Day 4 and 1.0 (0.3–1.7, 5.9) kg on Day 7. Weight loss on Day 4 was significantly greater in the high urinary salt excretion groups (Q3 and Q4) than in Q1 by Dunnett’s multiple comparison test (*P* = 0.018 and 0.004, respectively).

**Table 2:** Weight loss on Day 4 and Day 7 by quartiles of pre-hospital urinary salt excretion. ^a^*P* < 0.05 versus Q1. Abbreviations: IQR, interquartile range; Max., maximum; Min., minimum; Q, quartile.

Pre-hospital urinary salt excretion (g/day)	Day 4			Day 7		
	Median (IQR)	Min.	Max.	Median (IQR)	Min.	Max.
Total	0.7 (0.0–1.4)	−2.3	4.7	1.0 (0.3–1.7)	−1.8	5.9
Q1, 1.2–5.7	0.3 (−0.2–1.2)	−1.8	3.9	0.5 (0.03–1.38)	−1.8	3.0
Q2, 5.8–8.4	0.7 (0.03–1.3)	−1.5	4.0	0.9 (0.03–1.5)	−1.3	3.9
Q3, 8.5–11.3	0.9 (0.1–1.65)^a^	−0.9	4.7	1.1 (0.4–1.8)^a^	−1.0	5.4
Q4, 11.4–29.2	0.95 (0.2–1.53)^a^	−2.3	4.1	1.3 (0.4–2.13)^a^	−1.4	5.9

### Urinary salt excretion and serum sodium levels after admission

**Figure 2:**
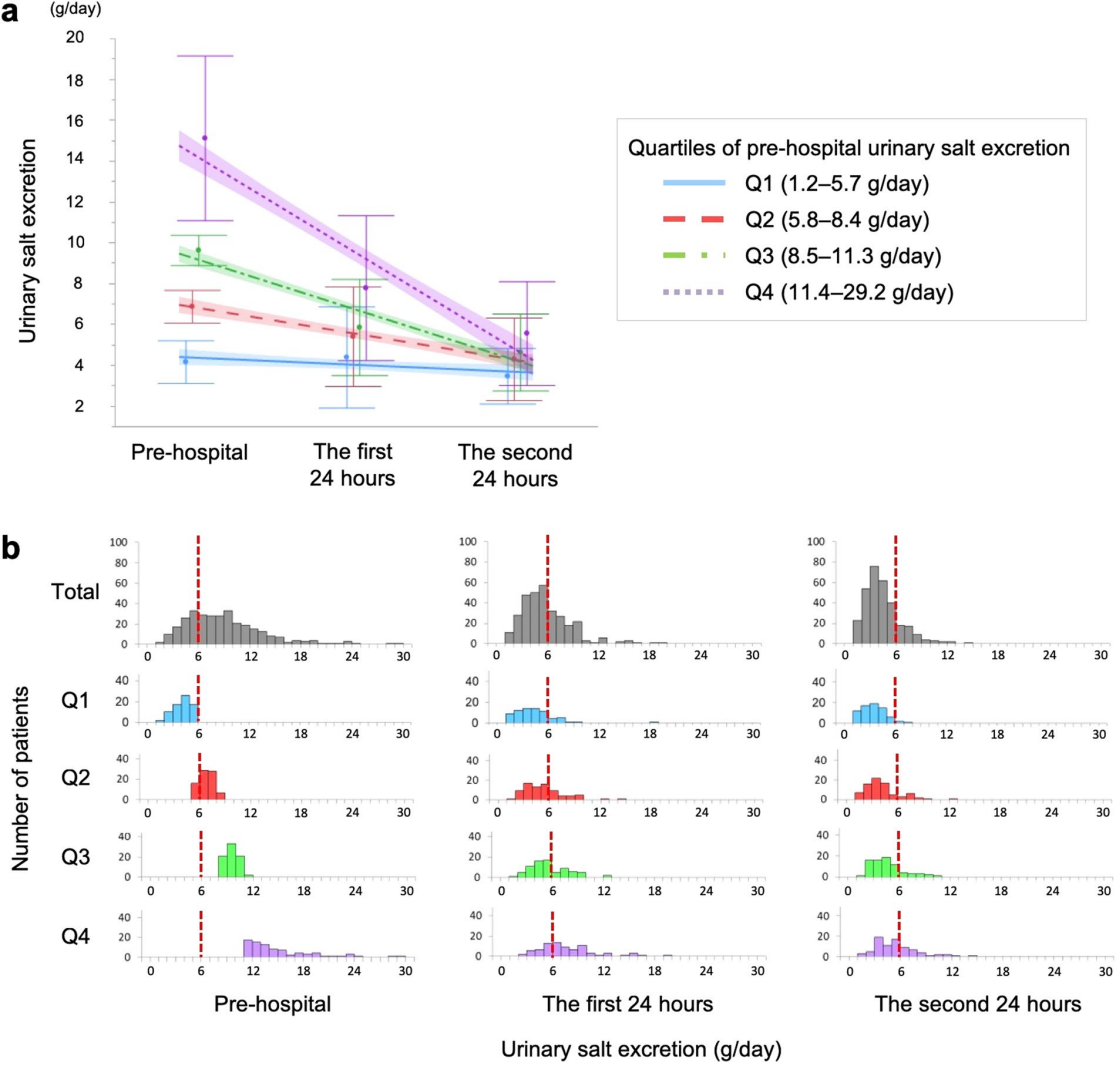
Changes in 24-hour urinary salt excretion after admission by quartiles of pre-hospital urinary salt excretion. (**a**) The 24-hour urinary salt excretion in all quartiles markedly decreased within 48 hours after admission. (**b**) Urinary salt excretion in all quartiles decreased to less than 6 g/day after admission. The dotted line indicates urinary salt excretion of 6 g/day. Abbreviations: Q, quartile.

The 24-hour urinary salt excretion in all quartiles markedly decreased within 48 hours after admission (Fig. 2a). The urinary salt excretion before, and in the first and second 24 hours after admission is shown in Fig. 2b. By consuming low-salt meals (5 g/day), the urinary salt excretion in all quartiles became less than 6 g/day after admission. However, 34.1% of patients in Q4 still had a urinary salt excretion of 6 g/day or more in the second 24 hours after admission, whereas only 4.2% in Q1 did (Table 3).

**Table 3:** Proportion of patients with urinary salt excretion of 6 g/day or more in the first and second 24 hours after admission by quartiles of pre-hospital urinary salt excretion. Abbreviations: Q, quartile.

Pre-hospital urinary salt excretion (g/day)	The first 24 hours	The second 24 hours
Total	37.7%	19.0%
Q1, 1.2–5.7	16.7%	4.2%
Q2, 5.8–8.4	30.0%	16.3%
Q3, 8.5–11.3	35.1%	19.5%
Q4, 11.4–29.2	65.9%	34.1%

Although serum sodium levels decreased significantly during hospitalization, they remained within the normal range for almost all participants (Table 4). The difference in serum sodium levels between Day 2 and Day 6 was not significant in Q2 to Q4 as compared with that in Q1.

**Table 4:** Serum sodium levels on Day 2 and Day 6 by quartiles of pre-hospital urinary salt excretion. Serum sodium levels decreased significantly during hospitalization in all quartiles. The difference in serum sodium levels between Day 2 and Day 6 was not significant in Q2 to Q4 as compared with that in Q1. Data are shown as the median (interquartile range). Abbreviations: Q, quartile.

Pre-hospital urinary salt excretion (g/day)	Serum sodium (mEq/L)		*P*-value	Difference (mEq/L)	*P*-value
	Day 2	Day 6			
Total	142 (140–143)	141 (139–142)	<0.001	1 (0–2)	
Q1, 1.2–5.7	142 (139–143)	141 (139–142)	0.001	1 (0–2)	Reference
Q2, 5.8–8.4	141 (140–143)	141 (139–142)	<0.001	0.5 (0–2)	0.72
Q3, 8.5–11.3	142 (141–143)	141 (140–142)	0.002	1 (0–1)	0.67
Q4, 11.4–29.2	141 (140–143)	141 (139–142)	0.014	0.5 (−1–2)	0.53

### Factors associated with rapid weight loss

To investigate factors associated with weight loss of 1 kg or more on Day 4, we conducted a multivariate logistic regression analysis (Table 5). In Model 1, which was adjusted for age and sex, body mass index (BMI) and urinary salt excretion in the first 24 hours were independently associated with rapid weight loss (adjusted odds ratio [OR], 1.11; 95% confidence interval [CI], 1.05–1.18 and adjusted OR, 1.15; 95% CI, 1.05–1.26, respectively). These factors remained significant even after adjustment for other factors: diuretic use, diabetes, proteinuria before admission, 24-hour systolic blood pressure, serum albumin, and serum creatinine (Model 2 and Model 3).

**Table 5:** Multivariate logistic regression analysis for weight loss of 1 kg or more on Day 4. Model 1: adjusted for age and sex. Model 2: adjusted for variables in Model 1 plus diuretic use, diabetes, and proteinuria before admission. Model 3: adjusted for variables in Model 2 plus 24-hour systolic blood pressure, serum albumin, and serum creatinine. Abbreviations: BMI, body mass index; CI, confidence interval; OR, odds ratio.

Variates	Unadjusted		Model 1		Model 2		Model 3	
	OR	95% CI	OR	95% CI	OR	95% CI	OR	95% CI
BMI	1.14	1.07–1.20	1.11	1.05–1.18	1.10	1.03–1.17	1.09	1.03–1.17
Urinary salt excretion in the first 24 hours	1.18	1.09–1.29	1.15	1.05–1.26	1.15	1.05–1.26	1.15	1.05–1.27
Pre-hospital urinary salt excretion	1.08	1.03–1.14	1.05	0.99–1.11	1.05	0.99–1.12	1.06	0.99–1.13

### Decreases in proteinuria after admission

**Figure 3:**
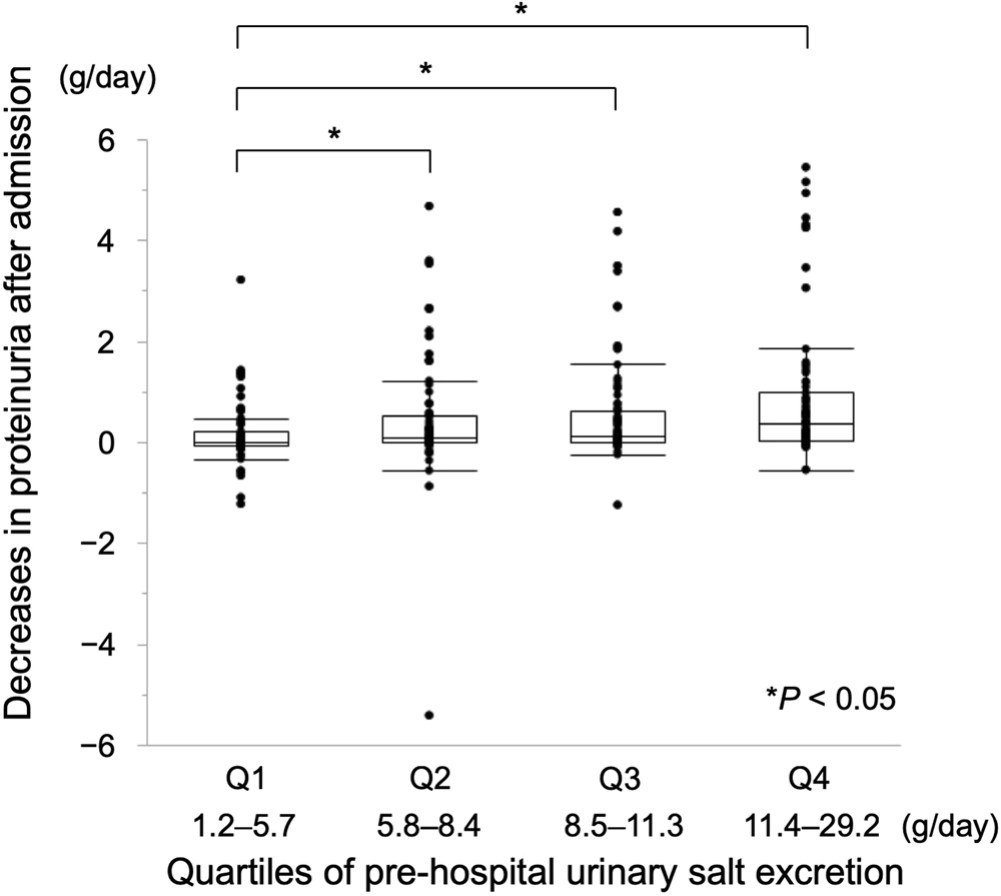
Decreases in proteinuria after admission by quartiles of pre-hospital urinary salt excretion. Proteinuria significantly decreased after admission in Q2 to Q4 compared with in Q1, and continued to decrease in the higher urinary salt excretion groups. Abbreviations: Q, quartile.

Decreases in proteinuria after admission, which were the secondary outcome, were compared among quartiles using Q1 as a reference group. Proteinuria decreased after admission significantly in Q2 to Q4 compared with in Q1 (Fig. 3).

### Urinary salt excretion and proteinuria after discharge

Lastly, changes in 24-hour urinary salt excretion and proteinuria after discharge were examined in a subset of 263 patients with 24-hour urine samples after discharge. The urinary salt excretion in the second 24 hours after admission and 3 months after discharge is shown in Supplementary Fig. S1. In all quartiles, only 20.2% exhibited urinary salt excretion of 6 g/day or more in the second 24 hours, but this percentage increased to 58.6% at 3 months after discharge (Supplementary Table S1). Increases in proteinuria after discharge were significantly higher in patients with urinary salt excretion of 6 g/day or more at 3 months after discharge (Supplementary Fig. S2).

## Discussion

This retrospective cohort study provided three important findings by investigating weight loss and decreases in proteinuria in CKD patients within 7 days of consuming a low-salt diet. First, many CKD patients exhibited rapid weight loss within a few days of consuming a low-salt diet during hospitalization. Second, weight loss with dietary salt restriction may be greater in CKD patients with a higher salt intake or higher BMI. Third, proteinuria can be decreased by dietary salt restriction in CKD patients with a high salt intake. These results were obtained using a population of CKD patients who participated in an in-hospital education program in Japan. In-hospital CKD education programs have been widely established under the public health insurance system in Japan, although the Ministry of Health recommends reducing the length of hospital stay. Each program consists of diet therapy, lectures on kidney disease, including renal replacement therapy options, and cardiovascular evaluation. Previous reports have examined the benefits of education programs^19–22^, but the purpose of our study was not to confirm their effects. Our subjects were selected because they underwent rapid changes in diet without additional medications, such as diuretics, which may affect weight loss. Therefore, the findings of this study can be applied to CKD patients hospitalized for any disease.

First, many CKD patients exhibited rapid weight loss within a few days of consuming a low-salt diet during hospitalization. Hung *et al*. reported that 52% of patients with stage 3–5 CKD have fluid retention as assessed by bioelectrical impedance analysis (BIA)^1^. Fluid retention is a major cause of hypertension in CKD patients and almost all CKD patients have hypertension at the beginning of dialysis. A previous study of pre-dialysis CKD patients with a mean eGFR of 14.5 ml/min per 1.73 m^2^ reported the prevalence of hypertension to be 95%^23^. Furthermore, uncontrolled 24-hour blood pressure is a risk factor for both end-stage renal disease (ESRD) and cardiovascular disease^24^. We did not evaluate changes in body fluid status with dietary salt restriction by specific methods like BIA, but as remarkable weight loss occurred within a few days after admission, the weight loss likely reflected reduced fluid retention more than a decrease in muscle or fat mass. A significant decrease in serum sodium levels during hospitalization, although clinically minor, suggested that a decrease in fluid volume occurred.

In this study, CKD patients with higher urinary salt excretion before admission demonstrated significant weight loss within a few days. In addition, these patients excreted more salt than they ingested even in the second 24 hours after admission. Vogt *et al*. reported that weight loss was significantly greater with a low-sodium diet (50 mmol/day) than with a high-sodium diet (200 mmol/day) in 6 weeks^13^. Similarly, Krinkken *et al*. reported that in normotensive healthy subjects, significant weight loss was observed with a low-salt diet (50 mmol/day) compared with a high-salt diet (200 mmol/day) in 7 days^16^. In contrast, de Brito-Ashurst *et al*. randomly provided a tailored low-salt diet or standard low-salt advice to hypertensive CKD patients and compared outcomes after 6 months. Compared with the standard low-salt advice, the tailored diet significantly reduced urinary sodium excretion (260 mmol/day to 103 mmol/day) and mean total body water (0.50 L), as assessed by BIA, but significant weight loss was not observed in either group^18^. In a meta-analysis including studies of varying periods from 2 weeks to 6 months, the authors were unable to conclude whether weight loss resulted from changes in body fluid status or fat mass because many studies did not describe changes in body fluid status or energy intake^17^. As we examined weight loss in a shorter period than that in those previous reports, we considered the weight loss within a few days to reflect body fluid reduction more than a decrease in fat or muscle mass.

Second, weight loss with dietary salt restriction may be greater in CKD patients with a higher salt intake or higher BMI. As approximately 90% of ingested salt is excreted in the urine^25,26^, it is useful to measure urinary salt excretion to estimate the daily salt intake^27^. In this study, urinary salt excretion in the first 24 hours after admission was independently associated with rapid weight loss immediately after dietary salt restriction. Evaluation of urinary salt excretion in the first 24 hours after admission can predict patients who are likely to lose weight due to abrupt dietary salt restriction. In addition, BMI was also independently associated with rapid weight loss due to dietary salt restriction in this study. Patients with abdominal obesity have an enhanced rate of tubular sodium reabsorption^28^. Moreover, higher BMI was reported to be associated with fluid retention during high salt intake in 78 healthy subjects^29^. Therefore, in the management of CKD patients admitted to the hospital, we can predict rapid weight loss within a few days based on high urinary salt excretion or high BMI.

Third, proteinuria can be decreased by dietary salt restriction in CKD patients with a high salt intake. In 95 healthy normotensive subjects, dietary salt restriction decreased urinary albumin excretion to within the normal range independent of blood pressure^30^. The same result was also reported for 34 CKD patients with a mean baseline proteinuria of 3.8 g/day^13^. Proteinuria is an independent risk factor for ESRD^31^ or all-cause and cardiovascular mortality^32^. Dietary salt restriction not only decreases blood pressure (BP), but also improves the antiproteinuric response to renin-angiotensin system inhibitors^33–35^. Furthermore, dietary salt restriction improves arterial stiffness and left ventricular diastolic function^2,4^. Thus, it is important to recommend dietary salt restriction for CKD patients with a high salt intake. Long-term prospective studies are required to clarify if continuation of dietary salt restriction leads to the sustained reduction of proteinuria.

There are some limitations in this study. First, as the timing of pre-hospital urine collection ranged from several weeks to several months before admission depending on the visit frequency, the pre-hospital urinary salt excretion did not reflect the salt intake just before admission. Urinary salt excretion in hypertensive patients is reported to increase in winter compared with that in summer^36^. Considering seasonal variations in dietary habits and physical activity, analysis using urine samples just before admission may be more ideal. As it is difficult to perform 24-hour urine collection at home in all cases, urine collection in the first 24 hours after admission may be practically useful. Second, our study lacked specific markers for fluid status such as brain natriuretic peptide (BNP) and BIA. BNP can reflect changes in body fluid status, but it is influenced by left ventricular abnormalities and accumulates in CKD patients^37^. Although it has been reported that fluid assessment by BIA is useful for CKD patients^1,38^, its utility has not been established. Even without using these expensive markers, short-term changes in body fluid status can be easily detected by daily measurement of body weight in clinical practice. Third, diuretics were discontinued in a few patients during hospitalization by decision of the attending physicians, but no patients started diuretics. Their impact on the results were considered to be limited because there was no significant difference in diuretic use or discontinuation among the quartiles of pre-hospital urinary salt excretion. Lastly, the study has the inherent limitations of a retrospective cohort study. Future randomized controlled trials comparing abrupt salt restriction with gradual salt restriction are needed for further investigation.

In conclusion, CKD patients with a high salt intake or high BMI exhibit rapid weight loss within a few days of consuming a low-salt diet during hospitalization. Dietary salt restriction is effective for reducing proteinuria in these patients, but long-term observation is needed to confirm the sustained effects.

## Methods

### Study design and participants

In this retrospective cohort study, we investigated weight loss in CKD patients during a 7-day hospitalization period while consuming a low-salt diet and clarified factors associated with rapid weight loss. We identified consecutive patients who participated in an in-hospital CKD education program between January 2012 and December 2014 at Omihachiman Community Medical Center, a tertiary care general hospital in Japan. We excluded patients who were missing data for body weight during hospitalization, or 24-hour urine samples before or during hospitalization. Patients with diseases unsuitable for dietary salt restriction, such as salt-wasting nephropathy or anorexia, were also excluded. The education program consisted of multidisciplinary patient education and cardiovascular evaluation for patients (Supplementary Fig. S3). During the 7-day hospitalization period, we served food that was low in salt (5 g/day) and protein (0.6–1.0 g/kg/day × ideal body weight based on individual CKD stages) according to the guidelines of the Japanese Society of Nephrology. Patients were permitted to stay out overnight from Day 4 to Day 5 to confirm nutritional therapy adherence.

This study was approved by the Ethics Committee on Human Research of Omihachiman Community Medical Center and was carried out in accordance with the Declaration of Helsinki. The requirement for informed consent was waived because of the retrospective design. Patient records/information were anonymized and de-identified prior to analysis.

### Outcomes and follow-up

The primary outcome was weight loss after admission. Body weight was measured immediately after admission and every morning from Day 2 to Day 7. Weight loss (kg) was calculated as the difference between body weight on each day from Day 2 to Day 7 and that on admission.

Secondary outcomes included changes in 24-hour urinary salt excretion and proteinuria after admission. Twenty-four-hour urine collection was conducted at the outpatient visit before admission and during the first and second 24 hours after admission (Supplementary Fig. S3). We evaluated the 24-hour urinary salt excretion before admission and in the first and second 24 hours after admission. The 24-hour urinary salt excretion (g/day) was calculated as the 24-hour urinary sodium excretion (mEq/day) divided by 17. We measured serum sodium levels on Day 2 and Day 6. Decreases in proteinuria were calculated as the difference in values between before admission and at the second 24 hours after admission.

In addition, 24-hour urinary salt excretion and proteinuria after discharge were assessed in a subset of patients who had 24-hour urine samples 3 months after discharge. Increases in proteinuria were calculated between the second 24 hours after admission and 3 months after discharge.

### Baseline assessment

Baseline characteristics of patients were extracted from electronic medical records as follows: age, sex, history of diabetes, and diuretic use. BMI was calculated as body weight (kg) divided by the height squared (m^2^). We collected baseline blood samples, and measured serum creatinine, serum albumin, and hemoglobin on Day 2. We used the following formula for Japanese patients with CKD to calculate the eGFR by gender: eGFR (ml/min per 1.73 m^2^) = 194 × serum creatinine^−1.094^ × age^−0.287^ (×0.739 if female)^39^. Patients wore a 24-hour ambulatory BP monitor (FB-270; Fukuda Denshi Co., Ltd., Tokyo, Japan) from Day 1 to Day 2. Ambulatory BP values were recorded every 30 minutes from 6:00 a.m. to 10:00 p.m. and every 60 minutes from 10:00 p.m. to 6:00 a.m., and the average was calculated as the 24-hour systolic and diastolic BP.

### Statistical analysis

Data are shown as numbers (percentage) for categorical variables and the median (interquartile range) for continuous variables. Patients were classified into quartiles based on pre-hospital urinary salt excretion. Weight loss after admission was compared among the groups by one-way ANOVA with post-hoc Tukey’s HSD tests. We compared weight loss among the groups on Day 4 and Day 7 by Dunnett’s multiple comparison test using the smallest quartile as a reference group. We compared the urinary salt excretion before and after admission in each group, and evaluated the proportion of patients with urinary salt excretion of 6 g/day or more in the first and second 24 hours after admission. We compared serum sodium levels on Day 2 and Day 6 using the Wilcoxon signed-rank test. We compared the difference in serum sodium levels between Day 2 and Day 6 by Dunnett’s multiple comparison test using the smallest quartile as a reference group.

We conducted a multivariate logistic regression analysis to clarify the effects of BMI and urinary salt excretion on weight loss in response to dietary salt restriction. In order to minimize the effects of changes in muscle and fat mass, and to more precisely evaluate changes in fluid status, we analyzed weight loss of 1 kg or more on Day 4. We also considered the subjects who stayed out overnight from Day 4 to Day 5. We used three logistic regression models because the model should be evaluated by several combinations of confounders for the sensitivity analysis. Model 1 was adjusted for age and sex, and Model 2 was adjusted for variables in Model 1 plus diuretic use, diabetes, and proteinuria before admission. Model 3 was adjusted for variables in Model 2 plus 24-hour systolic blood pressure, serum albumin, and serum creatinine. The results were expressed as an OR and its 95% CI.

We compared decreases in proteinuria after admission among the quartiles of pre-hospital urinary salt excretion by Dunnett’s multiple comparison test using the smallest quartile as a reference group.

In patients with 24-hour urine samples 3 months after discharge, we compared 24-hour urinary salt excretion before and after discharge in each group, and evaluated the proportion of patients with urinary salt excretion of 6 g/day or more in the second 24 hours after admission and at 3 months after discharge. We compared increases in proteinuria after discharge between patients with urinary salt excretion of 6 g/day or more and those with less than 6 g/day at 3 months after discharge by the Mann-Whitney test.

Differences were considered significant when the two-sided *P*-value was less than 0.05. Statistical analyses were performed using JMP software, Version 13 (SAS Institute Inc., Cary, NC).

## Supplementary information

Supplementary Information

## Data Availability

The datasets generated and analysed during the current study are available from the corresponding author on reasonable request.
