# Supplementary material for: Rapid weight loss with dietary salt restriction in hospitalized patients with chronic kidney disease
Source: Sci Rep. 2019 Jun 19;9:8787. doi: 10.1038/s41598-019-45341-6 (PMC6584671; doi:10.1038/s41598-019-45341-6)
Supplement: Supplementary file 1 — Supplementary Information [file 41598_2019_45341_MOESM1_ESM.pdf]

Supplementary Information

Rapid weight loss with dietary salt restriction in hospitalized patients with chronic kidney disease

Yu Mihara[1,2], Hiroshi Kado[1,2], Isao Yokota[3], Yayoi Shiotsu[1], Kazuhiro Sonomura[2], Tetsuro Kusaba[1], Tsuguru Hatta[2], Satoaki Matoba[4] & Keiichi Tamagaki[1*]

Affiliations:

[1]Department of Nephrology, Graduate School of Medical Science, Kyoto Prefectural University of Medicine, Kyoto, Japan.

[2]Department of Nephrology, Omihachiman Community Medical Center, Shiga, Japan.

[3]Department of Biostatistics, Graduate School of Medicine, Hokkaido University, Hokkaido, Japan.

[4]Department of Cardiovascular Medicine, Graduate School of Medical Science, Kyoto Prefectural University of Medicine, Kyoto, Japan.

Table of Contents

Figure S1 The 24-hour urinary salt excretion in the second 24 hours after admission and 3 months after discharge by quartiles of pre-hospital urinary salt excretion 3

Table S1 Proportion of patients with urinary salt excretion of 6 g/day or more in the second 24 hours after admission and 3 months after discharge by quartiles of pre-hospital urinary salt excretion 4

Figure S2 Increases in proteinuria between the second 24 hours after admission and 3 months after discharge 5

Figure S3 Schedule of the in-hospital CKD education program 6

Supplementary Figure S1. The 24-hour urinary salt excretion in the second 24 hours after admission and 3 months after discharge by quartiles of pre-hospital urinary salt excretion. The proportion of patients with urinary salt excretion of 6 g/day or more increased after discharge in all quartiles. The dotted line indicates urinary salt excretion of 6 g/day. Abbreviation: Q, quartile.

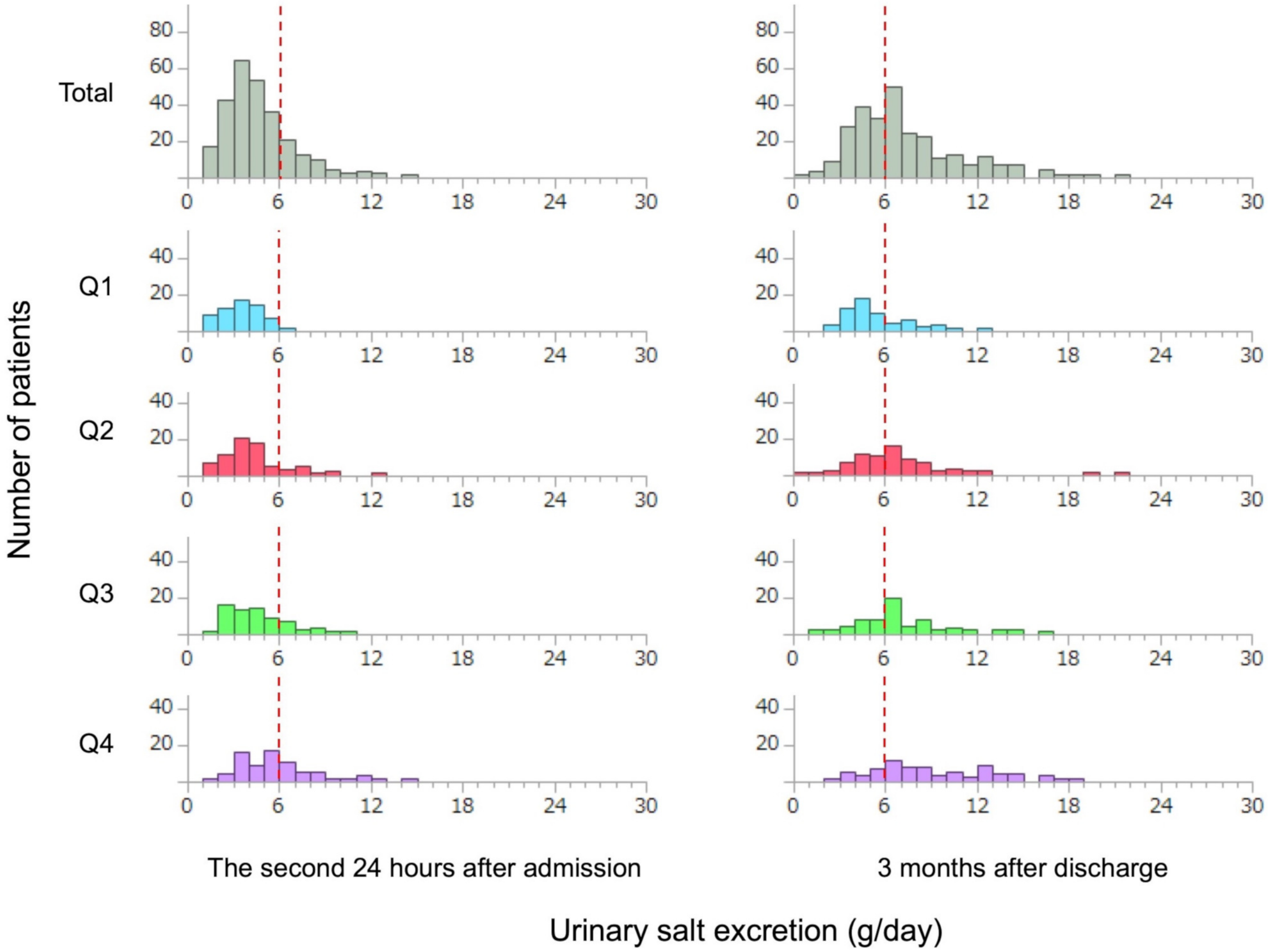

Supplementary Table S1. Proportion of patients with urinary salt excretion of 6 g/day or more in the second 24 hours after admission and 3 months after discharge by quartiles of pre-hospital urinary salt excretion. Abbreviation: Q, quartile.

Pre-hospital urinary salt excretion (g/day)	The second 24 hours	3 months after discharge
Total	20.2%	58.6%
Q1, 1.2–5.7	1.8%	28.1%
Q2, 5.8–8.4	16.9%	53.5%
Q3, 8.5–11.3	20.3%	60.9%
Q4, 11.4–29.2	38.0%	66.2%

Supplementary Figure S2. Increases in proteinuria between the second 24 hours after admission and 3 months after discharge. Increases in proteinuria were significantly higher in patients with urinary salt excretion of 6 g/day or more.

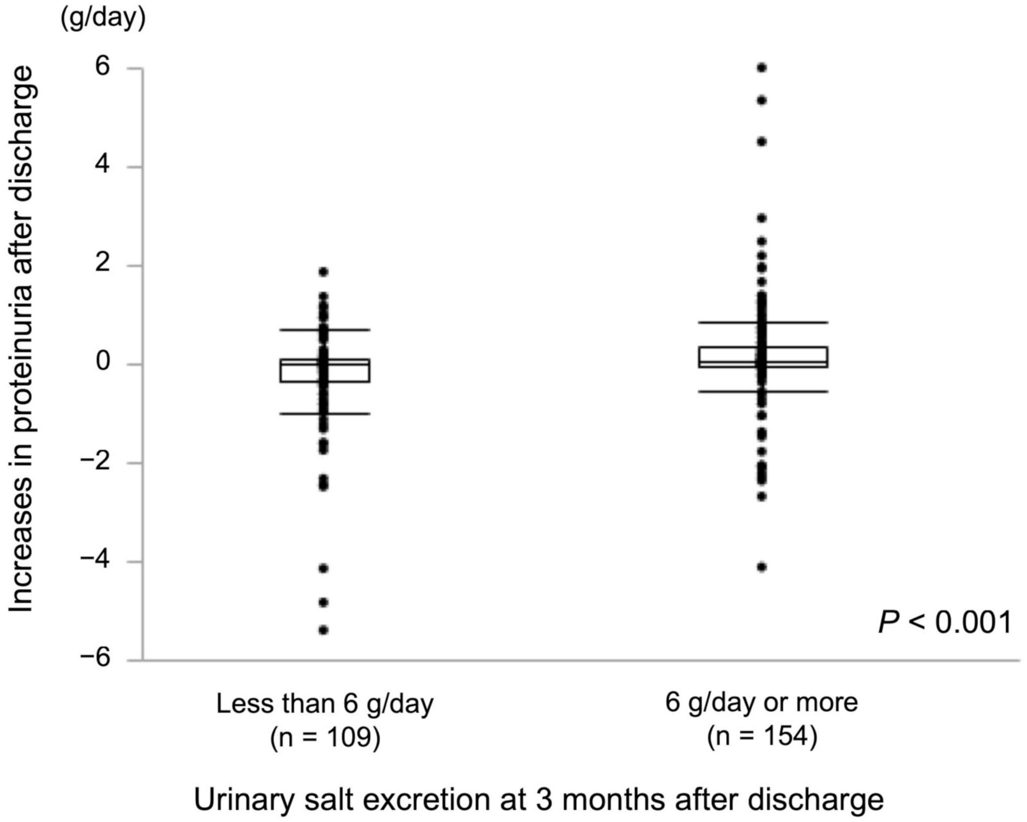

Supplementary Figure S3. Schedule of the in-hospital CKD education program. The education program consisted of multidisciplinary patient education and cardiovascular evaluation for the patients. During the 7-day hospitalization period, we served low-salt meals (salt content 5 g/day). Body weight was measured immediately after admission and every morning from Day 2 to Day 7. Twenty-four-hour urine collection was conducted during the first and second 24 hours after admission, and just before discharge. Patients were permitted to stay out overnight from Day 4 to Day 5 to confirm nutritional therapy adherence.

**Day**	**1**	**2**	**3**	**4**	**5**	**6**	**7**
Diet	Low-salt meals (salt content 5 g/day)						
Body weight	○	○	○	○	△	○	○
Nutritional counseling		○					○
Blood sampling		○				○	
24-hour urine sampling	⟷	⟷		Staying out overnight			
